# Comprehensive analysis of clinical, pathological, and genomic characteristics of follicular helper T-cell derived lymphomas

**DOI:** 10.1186/s40164-021-00224-3

**Published:** 2021-05-14

**Authors:** Sang Eun Yoon, Junhun Cho, Yeon Jeong Kim, Young Hyeh Ko, Woong-Yang Park, Seok Jin Kim, Won Seog Kim

**Affiliations:** 1 Division of Hematology-Oncology, Department of Medicine, Samsung Medical Center, Sungkyunkwan University School of Medicine, 81, Irwon-ro, Gangnam-Gu, Seoul, 06351 Korea; 2 Department of Pathology, Samsung Medical Center, Sungkyunkwan University School of Medicine, Seoul, Korea; 3 Samsung Genome Institute, Samsung Medical Center, Seoul, Korea

**Keywords:** Peripheral T-cell lymphoma-NOS, Angioimmunoblastic T-cell lymphoma, Follicular peripheral T-cell lymphoma, Nodal peripheral T-cell lymphoma with T follicular helper phenotype

## Abstract

**Background:**

The 2016 World Health Organization (WHO) classification introduced nodal lymphomas of T follicular helper (Tfh) cell origin, such as angioimmunoblastic T-cell lymphoma (AITL), follicular peripheral T-cell lymphoma (F-PTCL), and nodal peripheral T-cell lymphoma with T follicular helper phenotype (nodal PTCL with TFH phenotype). However, the accurate incidence rate and clinical characteristics of F-PTCL and nodal PTCL with TFH are unstudied.

**Methods:**

Between February 2012 to June 2020, a total of 207 cases diagnosed with nodal lymphomas of T follicular helper (Tfh) cell origin and PTCL-NOS were reviewed for clinical and histopathologic data. PTCL-NOS was defined to not correlate to any of the specific entities of mature T cell lymphoma in the WHO 2016 classification. We attempted to classify PTCL-GATA3 and PTCL-TBX21 by IHC staining. Target gene analysis was performed on a few patients with sufficient blood and tissue samples additionally.

**Results:**

Among 207 patients, 111 patients (53.6%) had AITL, 67 patients (32.4%) had PTCL-NOS, 19 patients (9.2%) had F-PTCL, and 10 patients (4.8%) had nodal PTCL with TFH phenotype. We re-defined and analyzed F-PTCL and nodal PTCL with TFH phenotype into other TFH lymphomas. AITL (N = 101/111, 91.0%) was found to have a higher frequency of stage III/IV cancers compared to other TFH lymphomas (N = 22/29, 75.0%) and PTCL-NOS (N = 53/67, 79.1%; *p*-value = 0.03). The OS of AITL and other TFH lymphomas was similarly superior to PTCL-NOS (*p*-value = 0.02). AITL and other TFH lymphomas showed the TBX21 subtype more commonly than the GATA3 subtype. Mutations related to the RAS family (*RHOA*) and those related to epigenetic regulators (*IDH2, DNMT3A*, and *TET2*) were shown mainly in AITL and other TFH lymphomas.

**Conclusions:**

Other TFH lymphomas appear to be a rare disease entity around one-quarter in nodal lymphomas of T follicular helper (Tfh) cell origin. Their less aggressive clinical feature than we did not expect is utterly different from PTCL-NOS and AITL. On the other hand, other TFH lymphomas share some characteristics, such as the cell of origin, a more common TBX21 subtype, and genetic variation such as RAS family mutation and epigenetic regulators, with AITL.

**Supplementary Information:**

The online version contains supplementary material available at 10.1186/s40164-021-00224-3.

## Background

Follicular helper T-cells (Tfh cells) are a subset of CD4+ T-cells that play two roles, serving as memory cells in the T-cell zone of lymphoid organs and effector T cells in areas of inflammation, depending on CCR7 homing chemokine expression [1, 2]. Normal Tfh cells, usually found in the germinal center of lymph nodes, produce IL21 and IL4; these stimulate B cells, suppress regulatory T cell differentiation, and lead to the proliferation of Tfh cells [3]. Previous studies have reported that the onset of Tfh-origin lymphoma in this normal immune process is caused by *RHOAG17V* mutation, which is involved in cell motility, adhesion, and cell‐cell interactions [4–7].

The 2016 World Health Organization (WHO) classification introduced nodal lymphomas of T follicular helper (TFH) cell origin through the expression of at least 2 or 3 TFH markers, including CD279/PD1, CD10, BCL6, CXCL13, ICOS, SAP, and CXCR5 [8]. It has been categorized into three different subtypes under the same umbrella, including angioimmunoblastic T-cell lymphoma (AITL), follicular peripheral T-cell lymphoma (F-PTCL), and nodal peripheral T-cell lymphoma with T follicular helper phenotype (nodal PTCL with TFH phenotype) [9]. Furthermore, PTCL-NOS was recently defined as excluding F-PTCL and nodal PTCL with TFH.

These three subtypes, AITL, F-PTCL, and nodal PTCL with TFH phenotype, were reported to share molecular abnormalities related to the same origin of Tfh cells [10–12]. Actually, the well-known mutation of AITL, such as *RHOA GTPase*, *TET2, DNMT3A, and IDH2*, was also reported in F-PTCL and nodal PTCL with TFH [13]. However, clinical features and therapeutic outcomes of these follicular helper T-cell-derived lymphomas have never been compared because these are relatively rare disease entities, especially F-PTCL and nodal PTCL with TFH. Furthermore, as the F-PTCL and nodal PTCL with TFH were established from the previous provisional disease entities, there are little data about their clinical behaviors and treatment outcomes. Therefore, we performed this comprehensive investigation of follicular helper T-cell derived lymphomas, including clinical, pathological, and molecular features of the Tfh cell origin lymphomas, and compared their treatment response and survival outcome with that of PTCL-NOS.

## Methods

### Study data collection and pathology review

By reviewing the all tissue sample at the diagnosis by hematopathologists from 2012, we collected 207 cases of AITL, F-PTCL, nodal PTCL with TFH, and PTCL-NOS from Samsung Medical Center among lymphoma cohort studies (the first cohort, NCT#01877109; the second cohort, NCT#03117036). Given that other TFH lymphomas’ rarity, we re-defined and analyzed F-PTCL and nodal PTCL with TFH phenotype into other TFH lymphomas. This study was approved by the Institutional Review Board of Samsung Medical Center (approval number. 2016-11-040-019). Written informed consent was obtained from each patient before study enrollment. It was conducted in accordance with the ethical principles of the Declaration of Helsinki and the Korea Good Clinical Practice guidelines.

Based on medical records, we gathered clinical information, including sex, age, complete blood count (CBC), direct antiglobulin test, indirect antiglobulin test, haptoglobin, immunoglobulin GAM, lactate dehydrogenase (LDH), beta-2 microglobulin (B2M), Eastern Cooperative Oncology Group (ECOG) performance status, bone marrow involvement, organomegaly, International Prognostic Index (IPI) [14], and Ann Arbor stage. We further collected the most used front-line chemotherapies, response rate, and survival outcomes according to subtype. The last patient registration was finished in December 2019, and the cut-off date for this study was June 2020.

### Pathologic review of T-cell lymphomas

All cases were reviewed by hematopathologists to differentiate AITL, F-PTCL, nodal PTCL with TFH, and PTCL-NOS according to the WHO 2016 criteria [2]. Three different subtypes under the same umbrella with the T-follicular helper (Tfh) phenotype were sorted through the presence of follicular dendritic cell (FDC) meshwork and follicular growth pattern by staining with CD21 and Tfh markers (CD4, PD-1, CXCL13, BCL6, and CD10). AITL was defined as the Tfh phenotype lymphoma with partial or total effacement of lymph node architecture and expanded FDC meshwork. Also, cases showing specific presentations in tissue samples such as, prominent high endothelial venules (HEVs) in paracortex, polymorphous inflammatory backgrounds containing histiocytes, plasma cells, and eosinophils, and expansion of follicular dendritic cell (FDC) meshwork, were classified as AITL. F-PTCL was characterized by a follicular growth pattern of Tfh-phenotype tumor cells lacking interfollicular involvement. The Tfh phenotype lymphoma without both FDC meshwork expansion and follicular growth pattern was classified as nodal PTCL with TFH (Additional file 1: Fig. S1a).

In addition, we performed immunohistochemistry (IHC) stains for T-bet, CXCR3, GATA3, and CCR4 on Formalin-Fixed Paraffin-Embedded (FFPE) tissue specimens obtained at the time of diagnosis. We tried to classify PTCL-GATA3 (T-helper 2 like origin) and PTCL-TBX21(T-helper 1 like origin) among follicular helper through these results T-cell origin lymphomas. PTCL-GATA3 type was defined when T-bet or CXCR3 were expressed more than 20%, vice versa PTCL-TBX21 type was defined as when GATA3 or CCR4 were expressed more than 50% without expression of T-bet and CXCR3 (Additional file 1: Fig S1b, Additional file 2: Table S1) [15–17].

### Blood sampling and targeted deep sequencing

Among 207 patients, target gene analysis was performed on 69 patients with sufficient blood at the time of examination for diagnosis. Whole blood samples were collected in Cell-Free DNA BCT tubes (Streck Inc., Omaha, NE, USA). After separation of plasma in the initial centrifugation, agranulocytes were separated by Ficoll gradient centrifugation and the granulocytes were separated from the bottom lymphocytes using RBC lysis buffer (Qiagen, Santa Clarita, CA, USA). Genomic DNA (gDNA) was isolated from granulocytes using a QIAamp DNA mini kit (Qiagen, Santa Clarita, CA, USA). Plasma DNA was obtained from 2 to 5 mL of plasma using a QIAamp Circulating Nucleic Acid Kit (Qiagen). The PBLs and plasma DNA libraries were created using a KAPA Hyper Prep Kit (Kapa Biosystems, Woburn, MA, USA) as described previously. In addition, capture baits for 66 genes selected from a 426 gene panel were customized and used for sequencing of cfDNA and their matched normal samples. After preprocessing, we identified somatic point mutations based on the previously reported iDES-enhanced CAPP-Seq with a minor modification [18]. The filtering steps to identify the variants were summarized as previously described [19].

### Tissue sample preparation

Target gene analysis was performed on tissues of 27 patients who collected plasma. An AllPrep DNA/RNA Mini Kit (Qiagen) was used to purify gDNA from formalin-fixed paraffin-embedded (FFPE) samples. The tumor biopsy sample libraries were constructed using the SureSelect XT reagent kit, HSQ (Agilent Technologies) according to the manufacturer’s instructions, and hybrid selection was performed using customized baits targeting 426 lymphoma-related genes. The filtering steps to identify the variants are summarized as previously described [20].

### Statistical analysis

Descriptive statistics were determined as proportions and medians, and the intergroup comparisons for categorical variables were assessed by the *X2* or Fisher’s exact test. The Kaplan–Meier method was used to evaluate progression-free survival (PFS) and overall survival (OS). PFS time was estimated as the time from diagnosis to the date of disease progression or death related to any cause. OS time was assessed as the time from diagnosis to the date of death or the last date of follow-up. All data were analyzed using the Statistical Package for Social Sciences software, version 24.0 (IBM Corp, Armonk, NY, USA).

## Results

### Characteristics description according to each subtype

Among 207 patients, AITL was the most common diagnosis (N = 111, 53.6%). Sixty-seven patients (32.4%) were diagnosed with PTCL-NOS, and the minority of patients had confirmed F-PTCL (N = 19, 9.2%) or nodal PTCL with TFH phenotype (N = 10, 4.8%). We have presented the clinical characteristics according to subtypes, such as AITL, other TFH lymphomas, and PTCL-NOS, in Table 1. Half of the patients with AITL (N = 66/111, 61.6%) and other TFH lymphomas (N = 15/29, 51.7%) were over 60 years old, compared to those with PTCL-NOS (*p*-value = 0.05). Stage III/IV disease (N = 176/207, 85.0%) at diagnosis was more common than stage I/II disease (N = 31/207, 15%). In particular, AITL (N = 101/111, 91.0%) was found to have a higher frequency of stage III/IV disease at diagnosis than other TFH lymphomas (N = 22/29, 75.0%) and PTCL-NOS (N = 53/67, 79.1%; *p*-value = 0.03). The percentage of patients evaluated with high-intermediate or high IPI groups (IPI ≥ 2) was 67.6% in AITL (N = 75/111), 37.9% in other TFH lymphomas (N = 11/29), and 50.7% in PTCL-NOS (N = 34/67; *p*-value = 0.01). Additionally, we tried to assess potential prognostic factors among a total of 207 patients with AITL, other TFH lymphomas, or PTCL-NOS. In both univariate and multivariate analysis, male, ECOG performance status ≥ 2, and thrombocytopenia were especially associated with inferior overall survival for the patients (Additional file 2: Table S2).

**Table 1:** Comparison of clinical characteristics according to subtype

Patients characteristics	Total	AITL	Other TFH lymphomas	PTCL-NOS	*p*-value
N (%)	207	111 (53.6)	29 (14.0)	67 (32.4)	
Age ≥ 60 years	99 (47.8)	66 (61.1)	15 (51.7)	27 (40.3)	0.05
Sex					
Male	132 (63.8)	64 (57.7)	16 (55.2)	52 (77.6)	0.01
Female	75 (36.2)	47 (42.3)	13 (44.8)	15 (22.4)	
ECOG					
0–1	182 (87.9)	95 (85.6)	24 (82.2)	63 (94.0)	0.15
> 2	25 (12.1)	16 (14.4)	5 (17.2)	4 (6.0)	
Stage					
I/II	31 (15.0)	10 (9.0)	7 (24.1)	14 (20.9)	0.03
III/IV	176 (85.0)	101 (91.0)	22 (75.9)	53 (79.1)	
IPI					
< 2	87 (42.0)	36 (32.4)	18 (62.1)	33 (49.3)	0.01
≥ 2	120 (58.0)	75 (67.6)	11 (37.9)	34 (50.7)	
B-symptom, presence	80 (38.6)	56 (50.5)	8 (27.6)	16 (23.9)	0.00
Hb < 10 g/dL)	43 (20.8)	31 (28.4)	2 (6.9)	10 (14.9)	0.01
Hemolytic anemia	9 (4.3)	9 (8.1)	0	0	0.00
Platelet < 100 k	36 (17.4)	20 (18.3)	0	16 (23.9)	0.01
Elevated LDH	119 (57.5)	71 (65.1)	10 (34.5)	38 (56.7)	0.01
Elevated B2M	107 (51.7)	65 (58.6)	7 (24.1)	35 (52.2)	0.00
IgG ≥ 1600 mg/dL	44 (21.3)	33 (29.7)	2 (6.9)	9 (13.4)	0.00
IgA ≥ 400 mg/dL	23 (11.1)	16 (14.4)	2 (6.9)	5 (7.5)	0.00
IgM ≥ 230 mg/dL	30 (14.5)	29 (26.1)	1 (3.4)	0 (0.0)	0.00
Serum EBV, positive	62 (30.0)	45 (40.5)	9 (31.0)	8 (11.9)	0.00
Splenomegaly	83 (40.1)	53 (47.7)	6 (20.7)	24 (35.8)	0.02
BM involvement	73 (35.3)	46 (41.4)	9 (31.0)	18 (27.7)	0.17

*ECOG* Eastern Cooperative Oncology Group, *LDH* lactate dehydrogenase, *B2M* beta-2 microglobulin, *EBV* Epstein-Barr virus; BM, bone marrow

Splenomegaly was more common in AITL (N = 53/111, 47.7%) compared to other TFH lymphomas (N = 6/29, 20.7%) and PTCL-NOS (N = 24/67, 35.8%; *p*-value = 0.02). Bone marrow involvement was demonstrated in 46 patients with AITL (41.4%), nine patients with other TFH lymphomas (31.0%) and 18 patients with PTCL-NOS (27.7%; *p*-value = 0.17). Elevated LDH was more common in AITL and PTCL-NOS compared to other TFH lymphomas (65.1% vs. 56.7%. vs. 34.5%; *p*-value = 0.01). Anemia was confirmed in 43 patients (20.8%), of which nine were hemolytic anemia reported only in patients diagnosed with AITL. Furthermore, hyper-paraproteinemia was mainly observed in AITL, and less frequently in other TFH lymphomas and PTCL. At the time of diagnosis, 38% of patients (N = 80/207) had B-symptoms, and the frequency of B-symptoms was the highest in AITL (N = 56/111, 50.5%); only 27.6% of patients presented with B-symptoms in other TFH lymphomas (N = 8/29). Thrombocytopenia (18.3% vs. 0% vs. 23.9%; *p*-value = 0.01), elevated B2M (58.6% vs. 24.1% vs. 52.2%.; *p*-value = 0.00), and serum EBV detection rate (40.5% vs. 31.0% vs. 11.9%; *p*-value = 0.00) were more common in AITL compared to other TFH lymphomas or PTCL-NOS.

### Treatment outcomes and survival outcomes related to each subtype

**Fig. 1:**
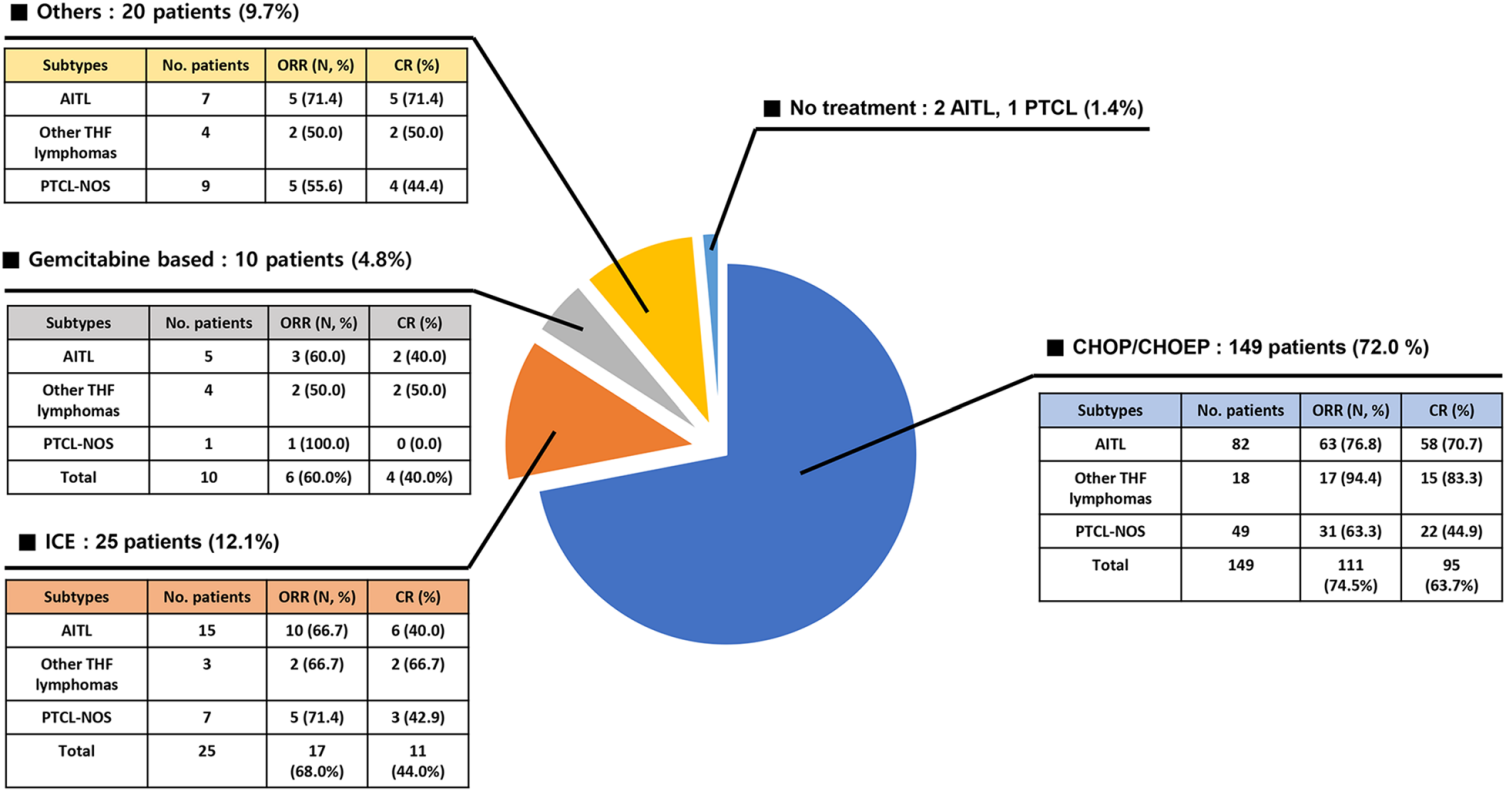
Overview of response rate according to front-line chemotherapy

Of 205 patients who received front-line chemotherapy, the majority of patients (N = 149/204, 72.0%) received CHOP-like (CHOP21, CHOP14, mini-CHOP) or CHOEP-like (EPOCH, DA-EPOCH, CHOEP) chemotherapy. Fewer patients received ICE (Ifosfamide, Mesna, Carboplatin, Etoposide) (N = 25/204, 12.1%) or gemcitabine-based chemotherapy (N = 10/204, 4.8%) as a front-line strategy. Three patients did not have an opportunity to receive chemotherapy due to fatal disease progression before starting treatment. Among 149 patients who received combined anthracycline chemotherapies, patients diagnosed with AITL achieved an ORR of 76.8% (N = 63/82) and a CR of 70.7% (N = 58/82), while those diagnosed with PTCL-NOS obtained an ORR of 63.6% (N = 31/49) and a CR of 44.9% (N = 22/49). Notably, patients diagnosed with other TFH lymphomas obtained a slightly higher ORR of 94.4% (N = 17/18) and CR of 83.3% (N = 15/18) compared to those diagnosed with AITL and PTCL-NOS (Fig. 1).

**Fig. 2:**
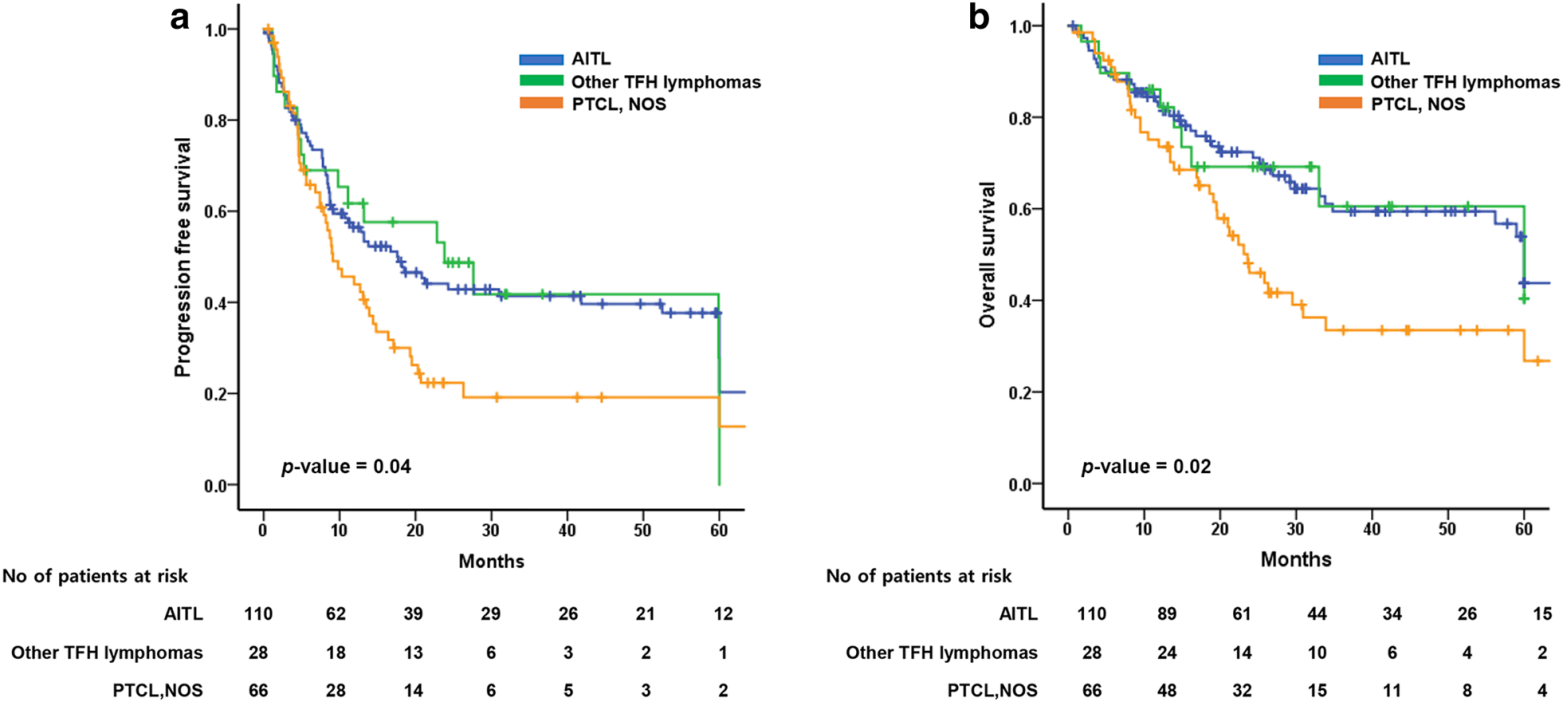
Comparison of progression-free survival (**a**) and overall survival (**b**) between AITL, other TFH lymphomas, and PTCL-NOS

When we compared PFS and OS according to AITL, PTCL-NOS, and other TFH lymphomas, we found that patients diagnosed with AITL and other TFH lymphomas had similar PFS (17.7 months, 95% CI 10.5–24.9; 23.8 months, 95% CI 6.3–41.3; 9.1 months, 95% CI 5.4–12.8, *p*-value = 0.04) and OS (60.0 months, 95% CI 55.8–64.2; 60.0 months, 95% CI 12.4–107.6; 12.6 months, 95% CI 18.5–28.7, *p*-value = 0.02), and these were superior compared to PTCL-NOS (Fig. 2).

### Analysis of molecular studies and immunohistochemistry analysis

**Fig. 3:**
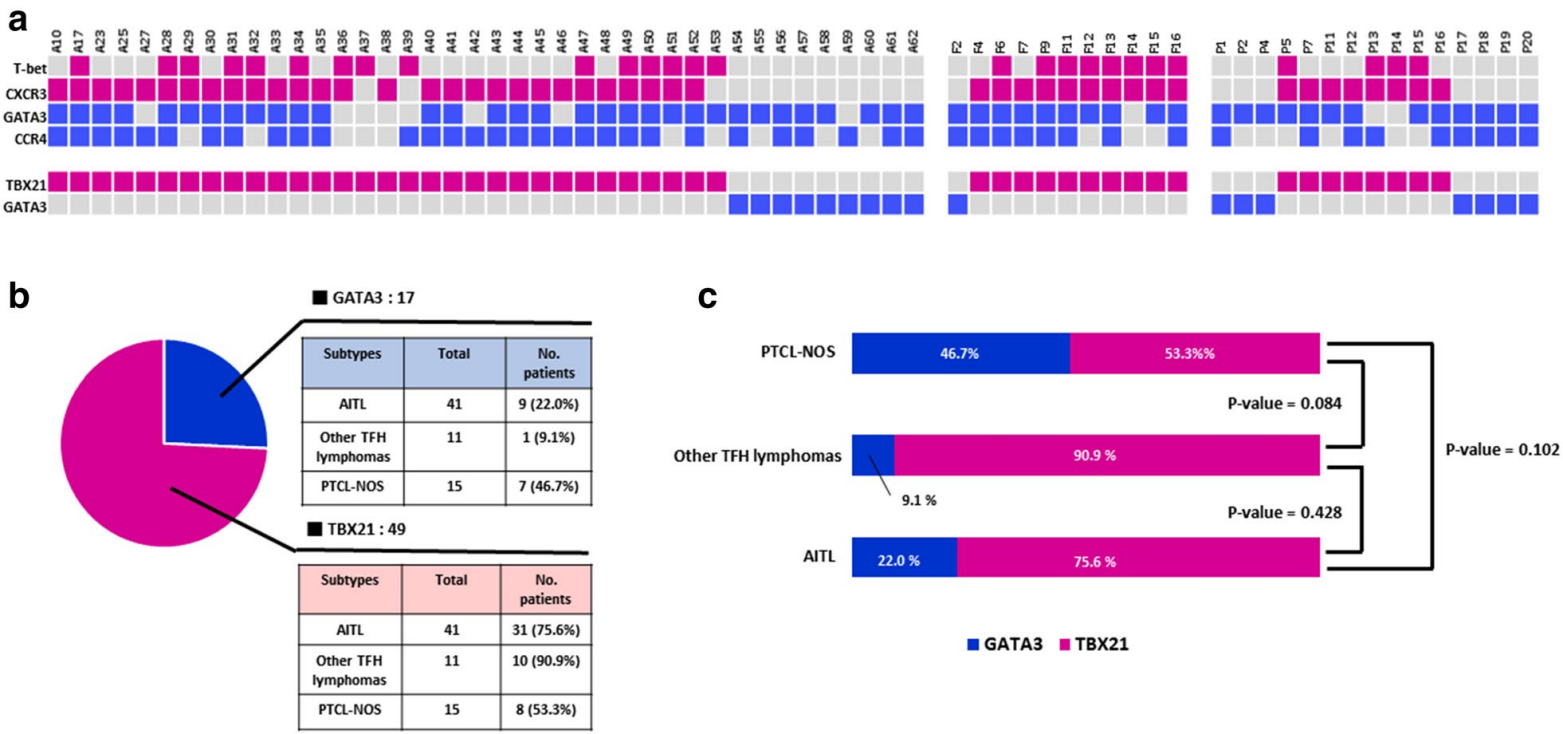
Overview of TBX21 and GATA3 staining results in AITL, other TFH lymphomas, and PTCL-NOS (**a**), the number of samples available for TBX21 and GATA3 staining (**b**), comparison of TBX21 and GATA3 expression among AITL, other TFH lymphomas, and PTCL-NOS (**c**)

**Fig. 4:**
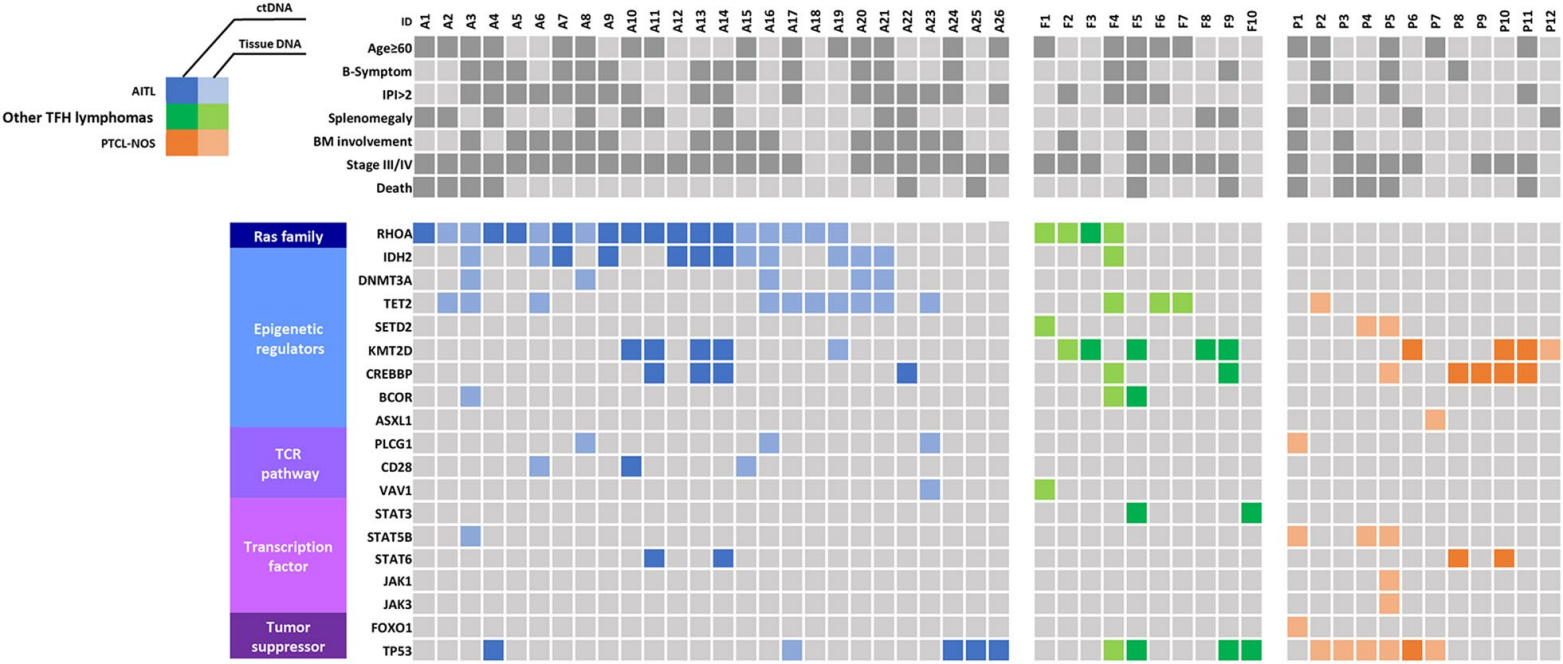
Overview of genetic expression and matched clinical information

Under the current guidelines, we performed IHC staining (T-bet, CXCR3, GATA3, CCR4) to estimate the proportion of TBX21 subtype and GATA3 subtype in 66 patients who had appropriate FFPE samples (Fig. 4a). Among 41 patients diagnosed with AITL, TBX21 was identified in 31 patients (75.6%), and GATA3 was described in 9 patients (22.0%). In other TFH lymphomas (N = 11), the TBX21 subtype (N = 10/11, 90.9%) was more common than the GATA3 subtype (N = 1/11, 9.1%). However, the proportion of TBX21 subtype (N = 8/15, 53.3%) and GATA3 subtype (N = 7/15, 46.7%) identified in PTCL-NOS showed no difference (Fig. 3b). However, we could not prove the significant difference of OS between patients with TBX21 and GATA3 (*p*-value = 0.15) from our findings (Additional file 1: Fig. S2). Comparing the staining results, the TBX21 subtype is more common in AITL and other TFH lymphomas than the GATA3 subtype. However, staining results did not demonstrate a significant difference between other TFH lymphomas and PTCL-NOS (*p*-value = 0.084), other TFH lymphomas and AITL (*p*-value = 0.428), or PTCL-NOS and AITL (*p*-value = 0.102; Fig. 3c).

In addition, we tried to describe genetic aberrations among AITL, other TFH lymphomas, and PTCL-NOS using blood and tissue samples collected at diagnosis through next-generation sequencing (NGS). Notably, mutations related to the RAS family (*RHOA*) and those related to epigenetic regulators (*IDH2, DNMT3A*, and *TET2*) were found mainly in AITL and other TFH lymphomas. There was no pattern of multiple *RHOA* presentations, and the most common *RHOA G17V* mutation was 20 cases, followed by 3 cases of *RHOA T19I*. However, mutations belonging to the TCR pathway, transcription factors, and tumor suppressors were found equally in AITL, other TFH lymphomas, and PTCL-NOS (Fig. 4). Finally, we attempted to confirm the association between IHC results and genetic alterations, but this was challenging due to the shortage of samples (Additional file 1: Fig. S3).

## Discussion

According to 2016 WHO classification, PTCL-NOS with a T-follicular helper (TFH) cell phenotype was re-defined into three subtypes, AITL, F-PTCL, and nodal PTCL with TFH, based on clinicopathologic features, immunohistochemistry (IHC), and typical genetic features [9]. However, due to the rarity of other TFH lymphomas, including F-PTCL and nodal PTCL with TFH, most studies retrospectively reported a pathologic review or molecular studies [13, 21, 22]. Thus, we could not precisely identify the clinical characteristics and disease progression patterns of other TFH lymphomas separated from AITL and PTCL-NOS. According to our retrospective data, other TFH lymphomas seems to present with less aggressive clinical features and has a favorable treatment response compared to AITL and PTCL-NOS. Moreover, the overall survival rate of other TFH lymphomas was similar to AITL and had better outcomes than PTCL-NOS. Although other TFH lymphomas was classified as PTCL-NOS with TFH cells, it expressed mainly T-bet and CXCR3, like PTCL-TBX21 (Th1 cell origin) [23]. As reported in other studies, the representative genetic alterations to induce a robust T follicular helper phenotype (*RHOA, IDH2, and DNMT3A*) of AITL also appeared in other TFH lymphomas [24]. However, other mutation profiles that play an essential role in T-cell receptor (TCR) signaling, transcription factors, and tumor suppressors were shared evenly between AITL, other TFH lymphomas, and PTCL-NOS [25].

PTCL-NOS includes all T-cell lymphomas with ambiguous pathological, clinical, and biological characteristics within the 2008 and 2016 WHO classifications. The International T-cell Lymphoma Project conducted a multicenter registry study to estimate the incidence of each subtype of PTCL worldwide. A diagnosis of PTCL-NOS was checked in 25.9%, and AITL was confirmed in 18.5% [26]. Furthermore, an Asian registry study also reported that PTCL-NOS represented 20.8% and AITL represented 24.7% of all cases [27]. Although there are differences in reported rates by each region, the incidence rate is around 20%. Since previous studies on the incidence rate of PTCLs were conducted based on the 2008 WHO classification, there was no mention of F-PTCL and nodal PTCL with TFH, which has started to attract attention based on the new 2016 WHO classification. We were able to re-review the pathology of 207 cases with PTCL-NOS and AITL and found 29 (14%) other TFH lymphomas cases through this study. Although our retrospective study could not represent all T-cell lymphoma entities, other TFH lymphomas is predicted to account for a very small number of T-cell lymphoma classifications, less than about 10% of PTCL-NOS. As such, the incidence rate of other TFH lymphomas is meager, and the search process is complex and challenging, requiring more careful attention during diagnosis.

Several studies have shown that survival outcomes of patients with PTCL-NOS are usually inferior compared to those with AITL. Several studies reported a 5-year overall survival (OS) rate of AITL patients ranging from 33 to 48% [28] and PTCL-NOS patients around 30% retrospectively [26, 29]. However, we found that the survival of PTCL-NOS excluding other TFH lymphomas were worse than that of AITL, while other TFH lymphomas and AITL had similar survival rates. Moreover, the CR rate of PTCL patients with standard anthracycline-based therapy showed ranges from 40 to 60%, according to previous studies [30, 31]. In this study, other TFH lymphomas demonstrated an excellent CR rate (about 80%) compared to PTCL-NOS (about 40%). Therefore, in previous studies, the PTCL-NOS is more likely to have included other TFH lymphomas, which have a better prognosis, and this likely overestimates the chemotherapy response rate and survival outcomes of PTCL-NOS. Therefore, PTCL-NOS needs to be classified in detail to obtain accurate survival and response rates in future registry studies or clinical studies targeting PTCLs.

PTCL-GATA3 exhibited T-helper 2 cell differentiation and presented genomic complexity with frequent loss or mutation of tumor suppressor genes targeting the *CDK2A/B-TP53,* and *PTEN-PIK3* pathways. PTCL-TBX21 manifested T-helper 1 cell differentiation and showed more mutations of cytotoxic effector genes and epigenetic regulator genes [32]. Heavican et al. showed that the mutation profile of PTCL-NOS with a TFH cell phenotype consisted of *TET2, DNMT3A,* and *RHOA*, which are common in AITL [33]. In our study, we also observed that other TFH lymphomas and AITL were mainly classified as PTCL-TBX21 based on IHC staining; these also exhibited *RHOA, IDH2,* and *DNMT3A*, which are included in the category of epigenetic modulators (Fig. 4). Thus, we suggest that other TFH lymphomas and AITL might show a better response rate to treatment with epigenetic modifiers and hypomethylating agents than PTCL-NOS. Paola et al. already conducted a retrospective study to show the efficacy of histone deacetylase inhibitors (HDACi) in PTCL-NOS with TFH versus non-TFH phenotypes. The response rate to HDACi in PTCL-NOS with a TFH cell phenotype was about twice as high as that of the non-TFH phenotype (56.5% vs. 29.4%, *p*-value = 0.0035) [34]. Therefore, our data suggest that new target agents under development or research for AITL might also be promising treatment strategies in other TFH lymphomas.

Interestingly, hypergammaglobulinemia was shown more in AITL, less in other TFH lymphomas [8]. In histopathologic findings, AITL is characterized by the proliferation of high endothelial venules and mixed inflammatory cell infiltration, which is not prominent in other TFH lymphomas [35, 36]. Hypergammaglobulinemia is caused by increased immunoglobulin production of polyclonal plasma cells, and this phenomenon can be considered a result of specific immunogenic stimulation of AITL. In other words, it can be assumed that the immunogenicity of tumor cells is one of the essential factors that make the clinical and pathological differences between AITL and other TFH lymphomas.

Through target sequencing research previously performed, we designed a customized panel of 66 genes targeting somatic mutations for various NHLs. Based on the research of longitudinal plasma samples using this panel, it was found that the sensitivity (88.0%) and specificity (> 99%) of the panel to detect somatic mutation were higher than that of tumor biopsies [19]. Same as the previous method, DNA was extracted and analyzed from separating each plasma, and blood cells, followed by germline DNA was filtered using DNA information from blood cells. Thus, it was available to consider the remained mutation as somatic mutations, not germline mutations. Thus, although blood and tissue samples were not obtained simultaneously in our study, we were able to compare the somatic mutation profile features between AITL, other TFH lymphomas, and PTCL-NOS based on the result that ctDNA well reflects the characteristics of the tumor tissue. Another limitation of our genetic data is that *DMNT2A* and *TET2* have a discrepancy between ctDNA and tissue samples. The first reason is the panel for tissue (425 genes) included more somatic mutation genes than that for ctDNA (66 genes). The second reason is *DMNT2A,* and *TET2 r*epresenting the clonal hematopoiesis of indeterminate potential (CHIP) did not include when we generated the ctDNA panel because these genes originated from neutrophils and generally filtered out due to low VAF. Although data related to somatic mutations did not represent the characteristics of AITL, other TFH lymphomas, and PTCL-NOS comprehensively, our findings were sufficient to show their tendency.

Given the rarity of the F-PTCL and nodal PTCL with TFH, we were unable to separate the two and analyze them as different diseases according to our findings. Although we integrated and analyzed the two diseases by other TFH lymphomas, we were able to observe the relative proportion, clinical features, and molecular characteristics of other TFH lymphomas. Consequently, it seems that other TFH lymphomas is a unique disease entity completely different from PTCL-NOS and AITL; however, it shares some characteristics like the cell of origin and genetic variation with AITL. Even though other TFH lymphomas is classified as a separate entity, PTCL-NOS remains a basket term inclusive of all unspecified entities within the 2016 WHO classification. Thus, further studies are continually needed to identify the specific disease characteristics of PTCL-NOS. We emphasize the importance of classifying other TFH lymphomas as a separate disease category from PTCL-NOS in future studies.

## Supplementary Information

**Additional file 1: Figure S1.** Methods to distinguish the T-follicular helper (Tfh) phenotype by CD21 and Tfh markers (CD4, PD-1, CXCL13, BCL6, and CD10) staining (a), methods to classify PTCL-GATA3 (T-helper 2 like origin) and PTCL-TBX21(T-helper 1 like origin) (b). **Figure S2.** Comparison of OS between PTCL-GATA3 (T-helper 2 like origin) and PTCL-TBX21(T-helper 1 like origin). **Figure S3.** Matching results of genetic information and TBX21/GATA3 staining.

**Additional file 2: Table S1.** Summary of antibody-related information used for TBX21-PTCL and GATA3-PTCL staining. **Table S2.** Univariate and multivariate analysis of PFS and OS for a total of 207 patients.

## Data Availability

All data will become publicly available upon request from the corresponding authors.

## Abbreviations

Tfh: T follicular helper
AITL: Angioimmunoblastic T-cell lymphoma
F-PTCL: Follicular peripheral T-cell lymphoma; nodal PTCL with TFH phenotype, nodal peripheral T-cell lymphoma with T follicular helper phenotype
ECOG: Eastern Cooperative Oncology Group
LDH: Lactate dehydrogenase
B2M: Beta-2 microglobulin
EBV: Epstein-Barr virus
BM: Bone marrow
