# Supplementary figures and images for: Comprehensive analysis of clinical, pathological, and genomic characteristics of follicular helper T-cell derived lymphomas
Source: Exp Hematol Oncol. 2021 May 14;10:33. doi: 10.1186/s40164-021-00224-3 (PMC8120779; doi:10.1186/s40164-021-00224-3)

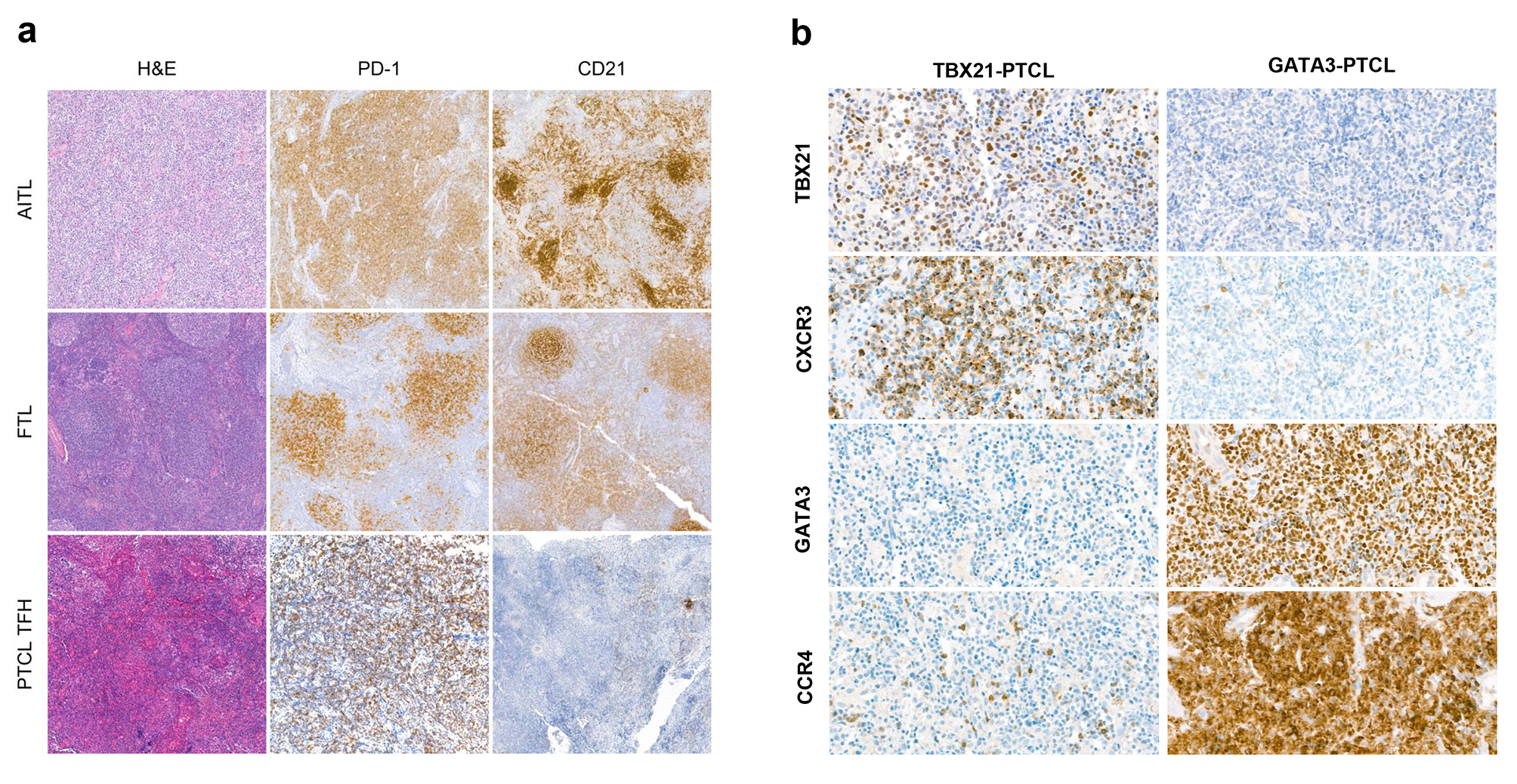

Supplement: Supplementary file 1 — Additional file 1: Figure S1. Methods to distinguish the T-follicular helper (Tfh) phenotype by CD21 and Tfh markers (CD4, PD-1, CXCL13, BCL6, and CD10) staining (a), methods to classify PTCL-GATA3 (T-helper 2 like origin) and PTCL-TBX21(T-helper 1 like origin) (b). Figure S2. Comparison of OS between PTCL-GATA3 (T-helper 2 like origin) and PTCL-TBX21(T-helper 1 like origin). Figure S3. Matching results of genetic information and TBX21/GATA3 staining. [file 40164_2021_224_MOESM1_ESM.zip › 40164_2021_224_MOESM1_ESM.tif]

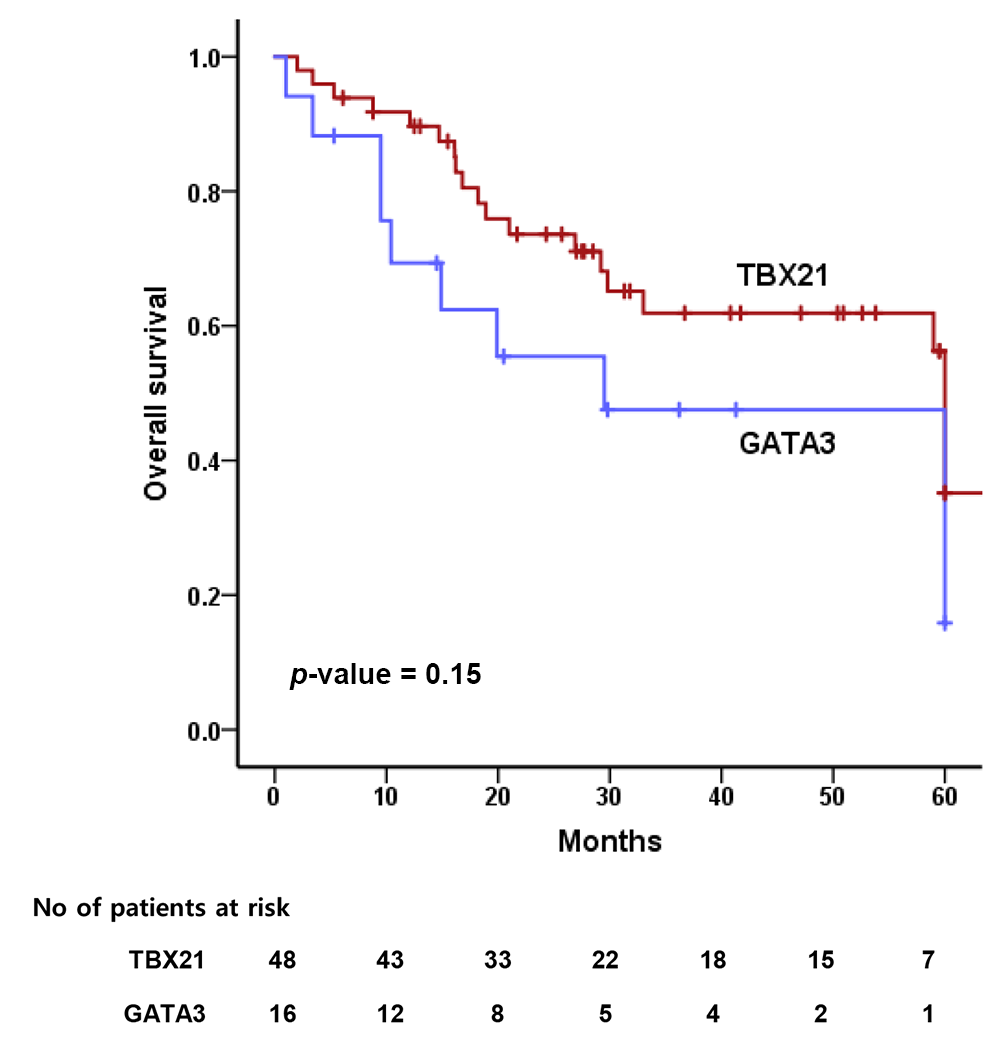

Supplement: Supplementary file 1 — Additional file 1: Figure S1. Methods to distinguish the T-follicular helper (Tfh) phenotype by CD21 and Tfh markers (CD4, PD-1, CXCL13, BCL6, and CD10) staining (a), methods to classify PTCL-GATA3 (T-helper 2 like origin) and PTCL-TBX21(T-helper 1 like origin) (b). Figure S2. Comparison of OS between PTCL-GATA3 (T-helper 2 like origin) and PTCL-TBX21(T-helper 1 like origin). Figure S3. Matching results of genetic information and TBX21/GATA3 staining. [file 40164_2021_224_MOESM1_ESM.zip › 40164_2021_224_MOESM2_ESM.tif]

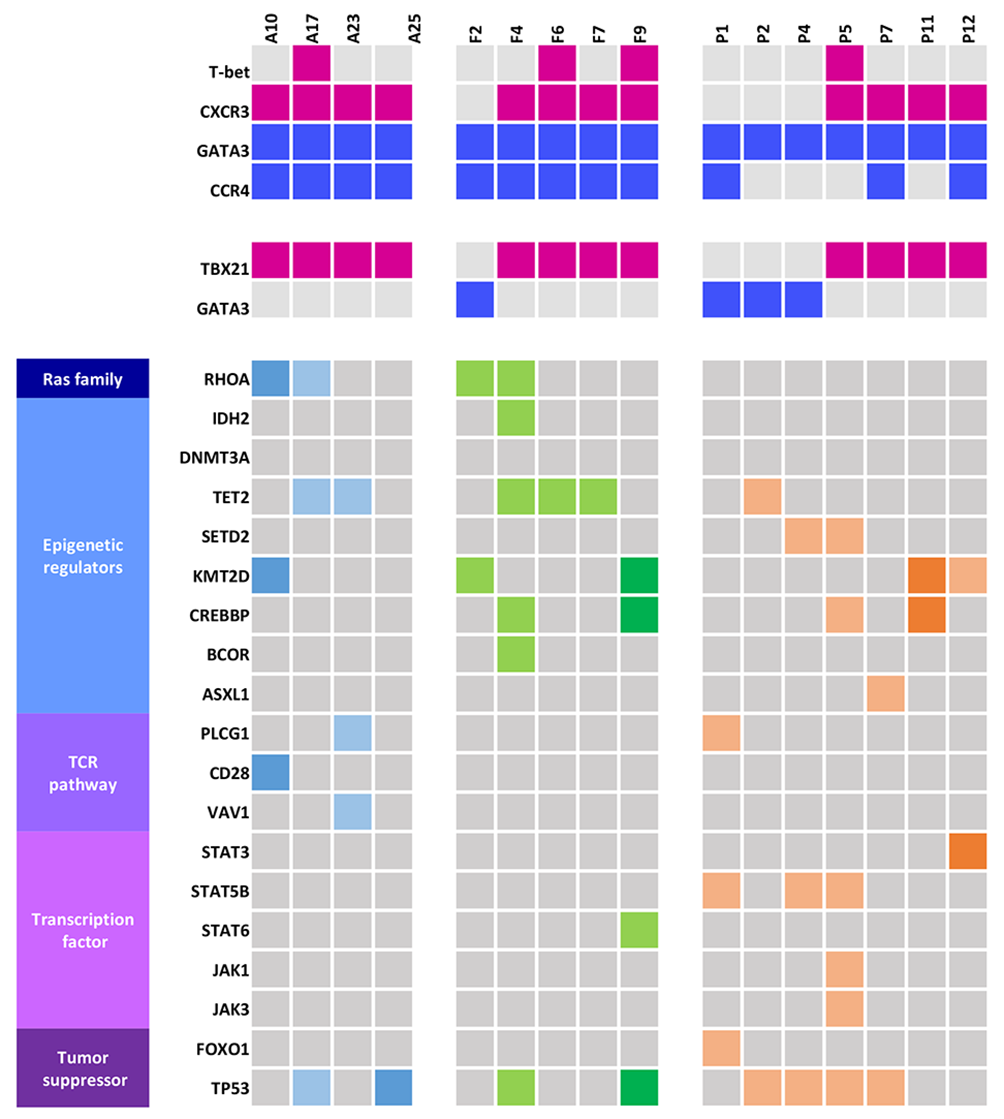

Supplement: Supplementary file 1 — Additional file 1: Figure S1. Methods to distinguish the T-follicular helper (Tfh) phenotype by CD21 and Tfh markers (CD4, PD-1, CXCL13, BCL6, and CD10) staining (a), methods to classify PTCL-GATA3 (T-helper 2 like origin) and PTCL-TBX21(T-helper 1 like origin) (b). Figure S2. Comparison of OS between PTCL-GATA3 (T-helper 2 like origin) and PTCL-TBX21(T-helper 1 like origin). Figure S3. Matching results of genetic information and TBX21/GATA3 staining. [file 40164_2021_224_MOESM1_ESM.zip › 40164_2021_224_MOESM3_ESM.tif]
