# Supplementary material for: Comprehensive analysis of clinical, pathological, and genomic characteristics of follicular helper T-cell derived lymphomas
Source: Exp Hematol Oncol. 2021 May 14;10:33. doi: 10.1186/s40164-021-00224-3 (PMC8120779; doi:10.1186/s40164-021-00224-3)
Supplement: Supplementary file 2 — Additional file 2: Table S1. Summary of antibody-related information used for TBX21-PTCL and GATA3-PTCL staining. Table S2. Univariate and multivariate analysis of PFS and OS for a total of 207 patients. [file 40164_2021_224_MOESM2_ESM.docx]

| **Name (clone)** | **Cat. no.** | **Manufacturer** | **Dilution** | **Antigen retrieval** | **Incubation time with primary antibody** | **Secondary and chromogen** |
| --- | --- | --- | --- | --- | --- | --- |
| Anti-T-bet / Tbx21 antibody [4B10] ab91109 | ab91109 | Abcam | 1:200 | 20 min with ER2 Buffer (pH 8.0) in 100℃ Bond-max | 15min with Bond-RX Autoimmunostainer  (Leica Biosystem, Melbourne, Australia) | 10 min Bond-RX Autoimmunostainer (Leica Biosystem, Melbourne, Australia) using Bond™ Polymer refine detection, DS9800 (Vision Biosystems, Melbourne, Australia) |
| GATA3(L50-823) Mouse Monoclonal Primary Antibody | 760-4897 | Ventana | RTU | 32min with CC1 in Ventana BenchMark XT | 16min with Ventana BenchMark XT in 37 ℃ | 12min with OptiView Amplification Kit (860-099) + OptiView DAB IHC Detection kit (760-700) |
| CCR4 Antibody | NBP1-86584 | NOVUS | 1:100 | 92min with CC1 in Ventana BenchMark XT | 60min with Ventana BenchMark XT in 37 ℃ | 12min with OptiView Amplification Kit (860-099) + OptiView DAB IHC Detection kit (760-700) |
| Purified Mouse Anti-Human D183 Clone 1C6/CXCR3 (also known as 1C6, LS177-1C6) (RUO) | 557183 | BD | 1:500 | 92min with CC1 in Ventana BenchMark XT | 60min with Ventana BenchMark XT in 37 ℃ | 12min with OptiView Amplification Kit (860-099) + OptiView DAB IHC Detection kit (760-700) |

**Table S1. Summary of antibody-related information used for TBX21-PTCL and GATA3-PTCL staining**

**Supplementary Table 2. Univariate and multivariate analysis of PFS and OS for a total of 207 patients.**

|  | Progression-free survival | | | | Overall survival | | | |
| --- | --- | --- | --- | --- | --- | --- | --- | --- |
| Variables | **Univariate** | **P-value** | **Multivariate** | **P-value** | **Univariate** | **P-value** | **Multivariate** | **P-value** |
| Age ≥ 60 years | 1.12 (0.8-1.6) | 0.50 |  |  | 1.94 (1.26-2.99) | <0.01 | 1.55 (0.88-2.71) | 0.12 |
| Female | 0.67 (0.48-0.98) | 0.04 | 0.83 (0.55-1.25) | 0.37 | 0.48 (0.30-0.78) | <0.01 | 0.52 (0.30-0.88) | 0.02 |
| ECOG ≥2 | 1.42 (0.86-2.33) | 0.17 |  |  | 3.38 (2.05-5.57) | <0.01 | 2.72 (1.47-5.01) | <0.01 |
| Stage III/IV | 1.61 (0.93-2.81) | 0.09 |  |  | 1.42 (0.73-2.73) | 0.30 |  |  |
| IPI ≥2 | 1.21 (0.86-1.72) | 0.28 |  |  | 1.77 (1.13-2.76) | 0.01 | 1.36 (0.65-2.88) | 0.42 |
| Presence of B Sx | 1.05 (0.74-1.48) | 0.78 |  |  | 1.42 (0.94-2.15) | 0.10 |  |  |
| Anemia | 1.63 (1.10-2.41) | 0.01 | 0.90 (0.52-1.57) | 0.71 | 1.56 (0.96-2.53) | 0.07 |  |  |
| Thrombocytopenia | 1.68 (1.11-2.56) | 0.02 | 1.59 (0.91-2.79) | 0.10 | 1.90 (1.18-3.09) | 0.01 | 2.42 (1.35-4.34) | <0.01 |
| Elevated LDH | 1.34 (0.95-1.89) | 0.10 |  |  | 1.76 (1.13-2.74) | 0.01 | 1.30 (0.66-2.54) | 0.45 |
| Elevated B2M | 1.89 (1.24-2.88) | < 0.01 | 1.51 (0.95-2.39) | 0.08 | 1.89 (1.12-3.18) | 0.02 | 1.05 (0.58-1.89) | 0.87 |
| Hypergammaglobulinemia | 0.88 (0.57-1.38) | 0.58 |  |  | 0.94 (0.54-1.63) | 0.81 |  |  |
| Splenomegaly | 1.09 (0.77-1.54) | 0.63 |  |  | 1.23 (0.81-1.86) | 0.34 |  |  |
| BM involvement | 1.75 (1.25-2.47) | <0.01 | 1.52 (0.99-2.33) | 0.05 | 1.50 (0.98-2.29) | 0.06 |  |  |
